# The neuromechanics of the soleus for fall prevention in aging

**DOI:** 10.3389/fphys.2025.1743559

**Published:** 2026-01-07

**Authors:** Jared R. Fletcher, Nicholas D. J. Strzalkowski

**Affiliations:** 1 Department of Health and Physical Education, Mount Royal University, Calgary, AB, Canada; 2 Department of Biology, Mount Royal University, Calgary, AB, Canada

**Keywords:** fall prevention, postural stability, balance, fall risk, tendon stiffness

## Abstract

Falls are a leading cause of injury-related hospitalization, morbidity, and mortality in older adults, with impaired postural control serving as a key predictor of fall risk. The triceps surae, and particularly the soleus, plays a central role in maintaining upright stance by generating continuous plantarflexion moments that stabilize the body’s center of mass. This mini-review summarizes evidence for the neuromechanical contributions of the soleus to postural stability and how these functions decline with age. Mechanically, the soleus acts as a brace for balance, providing sustained torque through fatigue-resistant type I fibers and a compliant Achilles tendon that buffers perturbations and contributes to ankle stiffness. Age-related reductions in tendon stiffness and rate of torque development compromise these stabilizing properties, increasing fall susceptibility. When passive stiffness is insufficient, the soleus compensates through active contraction, trading energy cost of activation for joint stability. Reflexively, the soleus serves as a stabilizer of balance through strong coupling to spinal, cutaneous, vestibular, and transcortical pathways that rapidly adjust muscle activation in response to perturbations. These reflex mechanisms also degrade with aging, leading to delayed, less adaptable responses. Together, age-related mechanical and neural deterioration reduce the soleus’ ability to sustain balance and contribute to fall recovery. Preserving soleus strength, tendon stiffness, and reflex adaptability through targeted neuromuscular and perturbation-based training may represent an underrecognized but effective strategy to mitigate fall risk and maintain postural control in older adults.

## Introduction

Impaired postural control is a major contributor to fall risk (Howcroft et al., 2017). Each year, 20%–30% of adults over 65 experience a fall, making falls the leading cause of injury-related hospitalization, morbidity, and mortality among Canadian seniors (Statcan, 2025). Between 2001 and 2019, fall-related mortality and hospitalization represented 87% of all injury-related hospitalizations in this population. The annual direct cost of injurious falls in Canada was estimated at CAD 5.6 billion in 2018 (Canada, 2025).

In quiet standing, the body’s center of mass (COM) lies anterior to the ankle joint, requiring a continuous plantarflexor (PF) moment to counteract forward sway. This moment arises from passive tissue properties, active muscle contractions, and reflex-mediated responses (Sasagawa et al., 2009). While the biarticular medial and lateral gastrocnemii contribute to both plantarflexion and knee flexion, the monoarticular soleus is mechanically specialized for sustained low-intensity plantarflexion moments during upright stance, making it uniquely suited to generate this continuous PF moment to maintain postural stability.

Among the PFs, the soleus contributes the largest proportion of active strength during quiet standing due to its large physiological cross-sectional area (Bohm et al., 2019) and high proportion of type I fibers (∼80%) (Edgerton et al., 1975; Johnson et al., 1973). This composition makes the soleus highly fatigue resistant, ideally suited for postural stability *maintenance* (Fitts and Holloszy, 1977). In contrast, the more mixed fibred gastrocnemii (∼57% type I) are better suited for intermittent, high-force reactive balance *corrections* (Héroux et al., 2014). The soleus therefore plays a larger role during quiet stance, while the gastrocnemii are critical to respond to unexpected balance perturbations. The soleus muscle-tendon unit also contributes passively to stability, with its short, slow fibers and long compliant Achilles tendon *might* buffer perturbations, allowing the muscle to remain isometric during small body sways. In contrast, with a stiffer tendon, any body sway would be sensed more directly by the muscle fibers themselves, since forces would be more directly transmitted to them. A greater tendon stiffness may also help maintain ankle stiffness (Loram et al., 2009). With aging, declines in tendon stiffness, together or independent of age-related declines in muscle strength, reduce PF force transmission to control postural stability (Stenroth et al., 2012; Miyazawa et al., 2025), increasing the risk of falls (Maki et al., 1994; Piirtola and Era, 2006). Preserving soleus strength and tendon stiffness may therefore help offset these mechanical deficits in older adults.

Neural pathways complement these mechanical features, providing rapid, reflexive modulation in response to internal and external perturbations (Chen and Zhou, 2011; Nakajima et al., 2006; Watson and Colebatch, 1998). The soleus is well suited for reflexive postural control due to its tonic activation, low recruitment threshold, and high reflex gain (Knikou, 2008). However, aging disrupts these reflex pathways, leading to delayed activation, diminished modulation, and reduced adaptability, all of which contribute to increased fall risk (Mynark and Koceja, 2002). Together, mechanical and reflexive mechanisms of the soleus may mediate postural sway to prevent these falls.

This mini-review summarizes current evidence for the neuromechanical contributions of the soleus to postural stability and fall prevention, with a focus on age-related declines. We examine the soleus’ key mechanical properties and neural control mechanisms that together support its role as a mechanical brace and reflexive stabilizer. The neuromechanics of quiet stance do not fully generalize to real-world slips and trips; however, linking soleus characteristics to fall risk, we highlight the soleus as a promising target for interventions aimed at preserving postural control in older adults.

## Soleus as a mechanical brace for balance

PF strength and power are both linked to fall risk in aging. Older fallers exhibit lower PF strength, reduced rate of torque development (RTD), and diminished impulse compared to non-fallers (LaRoche et al., 2010). Static balance, however, is only weakly correlated with PF strength, whereas dynamic balance demonstrates a stronger relationship (Tavakkoli et al., 2021). Han and Yang (2015) demonstrated that maximal joint power predicted slip outcomes with greater accuracy than maximal joint torque, suggesting that the ability to generate power, particularly across larger, more proximal joints like the hip and knee, is more relevant to slip recovery than maximal strength.

The anterior moment of the body’s centre of mass cannot be counteracted by passive ankle stiffness alone; muscles must remain active to maintain postural stability. The fatigue-resistant Type I fibers of the soleus are well-suited for this maintenance of force, particularly during aging when muscle power declines to a greater extent than muscle strength (Wiegmann et al., 2021). Muscle strength correlates with Achilles tendon stiffness, which also declines with age, reducing the passive contribution of tendon stiffness to postural stability with aging. Reduced Achilles tendon stiffness slows RTD, lengthens electromechanical delay (Isabelle et al., 2003; Muraoka et al., 2025), and can shift fibers away from their optimal length potentially reducing maximal force output (Bohm et al., 2019). The cumulative effect is slower mechanical responses during standing balance, and an impaired ability to generate the rapid torque needed for recovery from slips or trips. Together, compared to the gastrocnemius with its relatively high proportion of Type II fibers, muscle quantity (Ishihara et al., 1987; Deschenes, 2004), quality (Kim and Franz, 2021) and motor unit number (Dalton et al., 2008) are relatively preserved in aging in the soleus making it equipped to *prevent* falls in older adults.

Although our focus is quiet stance, gait mechanics highlight how tendon-fascicle characteristics shape energetic cost and torque capacity. In healthy, recreationally active young males walking at their preferred walking speed, the soleus undergoes a moderate stretch-shorten cycle across the shallow ascending limb of its force-length relationship (Rubenson et al., 2012). With reduced Achilles tendon stiffness, muscle fascicles shorten more against a more compliant in-series tendon. In theory, this additional shortening would require an additional muscle energy cost (Fletcher et al., 2013). However, during gait, much of the soleus fascicle shortening occurs during deactivation (Rubenson et al., 2012), while the fascicles are still at a relatively high (∼75%) maximal force potential. This stretch-shorten fascicle behaviour appears reduced when older adults walk at the preferred speed of young adults. When older adults walk at the preferred speed of young adults (approximately 20% faster than older adults), the stretch-shorten cycle behaviour of the soleus fascicles is reduced, and older adults walk with shorter fascicle lengths at this speed (Panizzolo et al., 2013). The shorter fascicle lengths would reduce the soleus force potential at that speed, potentially elevating the metabolic cost of contraction and/or the fatiguability of the soleus during walking (Beck et al., 2022; Skaper et al., 2025; Nguyen et al., 2025). To compensate, older adults typically choose a slower preferred walking speed such that the soleus fascicles undergo a greater stretch-shorten cycle and/or operate at shorter muscle lengths comparable to young adults (Panizzolo et al., 2013). Together, reduced tendon stiffness, slower RTD, and diminished passive joint stiffness compromise rapid balance corrections, even as the soleus remains the primary contributor to PF moment during upright stance.

### Active compensatory role

When passive contributions to ankle joint stiffness are insufficient (e.g., with aging), the neuromuscular system compensates through increased muscle co-contractions. Sasagawa et al. (2009) demonstrated that diminished passive stiffness at the ankle leads to higher levels of co-contraction between PFs and dorsiflexors. While this strategy stabilizes the joint, it might come at a higher energy cost of walking (Piche et al., 2022; Peterson and Martin, 2010), since a larger volume of agonist-antagonist muscle must be recruited (Taylor, 1994).

The notion that co-contraction is a compensatory mechanism to an insufficient and/or reduced passive stiffness as a neuromuscular strategy to aid in fall prevention is evident in clinical hypermobility and in aging. For example, in individuals with joint hypermobility-related conditions, passive joint stiffness is reduced as a result of changes in collagen content and cross-linking. Co-contraction of lower limb joints appears as an active muscle solution to this reduced passive joint stiffness. For example, Sheehan et al. (2025) show that co-contraction of agonist-antagonist ankle joint pairs during walking is greater in individuals with joint hypermobility, compared to age- and sex-matched individuals without joint hypermobility. Similarly, in older adults, Pijnappels et al. (2001) found significant increases in the activity of the hamstrings, gastrocnemius, soleus, and gluteus maximus following a trip perturbation compared to normal walking. Co-contraction of muscles crossing the ankle joint were higher in older compared to younger adults (Iwamoto et al., 2017), while within an older adult cohort, Nelson-Wong et al. (2012) demonstrated a higher co-contraction ratio in older adults deemed at a greater risk of falls. These findings indicate that co-contraction strategies extend beyond the ankle to involve multiple muscle groups in the lower limb, highlighting the systemic nature of neuromuscular compensation for reduced passive stiffness. The fatigue-resistant soleus can remain active for long periods of time, whereas its primary antagonist, the tibialis anterior, fatigues more readily. This asymmetry may influence how long co-contraction can be sustained, reducing the overall efficiency of postural control.

Collectively, these results highlight the cost-benefit of active muscle co-contraction to stabilize joints and prevent falls. Co-contraction provides mechanical stability when passive structures are insufficient to maintain stability but an increase in metabolic cost associated with muscle activation may accelerate muscle fatigue. Because tibialis anterior fatigues more readily than the soleus, sustained co-contraction may degrade dorsiflexor output over time. The soleus’ passive and active mechanical properties make it uniquely effective as a stabilizer for balance while mitigating PF fatigue when passive stiffness is insufficient to maintain postural control.

### Soleus as a reflexive stabilizer of balance

The soleus plays a critical role in postural control and is uniquely positioned as a reflexive stabilizer during quiet stance because of its slow-twitch phenotype, continuous postural activation, and strong spinal connectivity (Edgerton et al., 1975; Knikou, 2008). Soleus activity and tone are shaped by multiple reflex pathways that help it anticipate and respond to balance perturbations. The soleus is commonly included in postural reflex studies as it provides a reliable window in reflex function across the life span. The spinal stretch reflex, commonly studied through the H-reflex, provides a measure of motor neuron excitability (Knikou, 2008). Cutaneous afferent feedback from the foot sole modulates soleus activity to support upright stance (Zehr and Stein, 1999), while vestibulo-spinal reflexes drive soleus activity to orient the body in space (Fitzpatrick and Day, 2004). In addition, longer-latency transcortical feedback loops provide context-dependent corrections to soleus activity (Sinkjær et al., 1999). In response to a balance perturbation, long-latency reflexes (∼120–150 m) dominate postural corrections, whereas contributions from short-latency spinal reflexes are comparatively small (Payne et al., 2021; Willaert et al., 2024). These reflex pathways highlight the soleus as an important reflexive stabilizer, and one that shows deterioration with aging (Mynark and Koceja, 2002), and modulation with training interventions (Taube et al., 2008). While these reflex pathways contribute to postural control during quiet stance, their direct impact on fall-recovery remains uncertain.

#### The spinal stretch and H-reflex

Short-latency stretch reflexes provide rapid response for postural corrections. During quiet stance, forward sway stretches the soleus, increasing muscle spindle Ia activity that monosynaptically enhances PF activity (Chen and Zhou, 2011). These reflexes continuously modulate motor neuron excitability and stabilize the body. Compared to the gastrocnemius, the soleus contains a higher density of muscle spindles (Kissane et al., 2022), reinforcing its role as an important reflexive stabilizer.

Importantly, greater reflex amplitudes are not indicative of better balance. Reflex gains decrease when moving from sitting to standing, reflecting task-dependent modulation (Zehr and Stein, 1999). Individuals with excellent balance, such as trained dancers or gymnasts, often show reflex suppression and modulation during standing (Nielsen et al., 1993). Under postural threat however, reflex gains often increase, interpreted as adaptive stiffening to improve stability (Horslen et al., 2018). Together, these findings suggest that reflexive balance control relies on both stretch reflex sensitivity and the capacity to appropriately scale reflexes based on task demands.

The H-reflex (Hoffman reflex) is the electrical analogue of the mechanical stretch reflex and is one of the most widely studied human reflexes (Knikou, 2008). In the soleus, it is elicited by stimulating the tibial nerve, with amplitudes reflecting the efficacy of Ia input onto alpha motor neurons. H-reflex gain is modifiable through training and sensory context (Taube et al., 2008; Thompson et al., 2009), and in older adults, where reduced H-reflex gain has been observed alongside balance improvements (Mynark and Koceja, 2002).

Aging is associated with reduced H-reflex amplitude, diminished modulation across postures, and weaker adaptation to destabilizing conditions (Mynark et al., 2001; Alizadehsaravi et al., 2020). It is suggested that older adults rely more on central than peripheral mechanisms to control soleus activation (Klass et al., 2011). These changes may reflect impaired spinal integration and contribute to elevated fall risk, though causal links to functional balance remain unclear.

#### Cutaneous reflexes

Cutaneous afferents from fast- and slow-adapting mechanoreceptors in the foot sole and dorsum provide contact pressure, shear force, and velocity feedback that reflexively modulates soleus motor neuron excitability (Zehr and Stein, 1999; Strzalkowski et al., 2018). They exhibit strong synaptic coupling with motor neurons innervating the soleus and other leg muscles (Fallon et al., 2005). Soleus reflexes are stronger and more consistent than those in the gastrocnemii, reflecting its continuous role in the control of standing balance (Zehr and Stein, 1999).

Soleus cutaneous reflexes are location- and context-dependent. For example, electrical stimulation of the heel has been shown to facilitate soleus activity, while forefoot stimulation produces inhibition (Nakajima et al., 2006). Reflex gain is reduced during standing compared to sitting, consistent with task dependent modulation. During gait, foot sole stimulation elicits inhibitory responses that guide foot placement, highlighting the role of cutaneous input in dynamic balance (Zehr et al., 2014).

Aging reduces both cutaneous sensitivity and reflex amplitude decline, potentially due to peripheral receptor loss, impaired central processing, and reduced integration with motor outputs. Mechanically evoked cutaneous reflexes are detectable in nearly all young adults, but in just over half of older adults, with significantly lower reflex gain and coherence in the older group (Peters et al., 2016a). This cutaneous reflex degradation may limit the soleus’ ability to adaptively respond to surface changes or slips, increasing the risk of falls. Declines in both cutaneous and proprioceptive sensitivity are also associated with poorer balance control in healthy older adults (Song et al., 2021).

#### Vestibulospinal reflexes

Vestibular input plays a central role in the maintenance of postural orientation by encoding head acceleration and position with respect to gravity. This sensory feedback drives postural muscle activity in axial and limb muscles, including the soleus, to stabilize the body during standing and gait (Fitzpatrick and Day, 2004). Among the lower limb muscles, the soleus shows robust, directionally specific responses to vestibular stimulation, reinforcing its role as a reflex stabilizer (Ali et al., 2003).

Electrical vestibular stimulation (EVS) is commonly used to noninvasively probe the vestibulospinal contributions to balance. During upright stance, EVS evokes short- and medium-latency reflexes in soleus, and other postural muscles that are phase-locked to the EVS waveform and the head position and modulated by stance width and vision (Fitzpatrick and Day, 2004). EVS-evoked soleus responses reflect the functional integration of vestibular input with ongoing postural commands and are sensitive to task demands and biomechanical context.

Like other reflex pathways, soleus vestibulospinal reflexes deteriorate with age. Older adults show reduced vestibulospinal reflex amplitudes and altered vestibular perceptual thresholds, including both weaker descending drive and impaired sensory integrations in response to EVS (Dalton et al., 2014; Peters et al., 2016b). These deficits are associated with diminished ability to recover from unexpected perturbations and likely arise from degraded central processing within vestibular and postural control networks. Subthreshold EVS can improve postural stability in older adults, possibly by enhancing vestibular-somatosensory integration and recalibration (McLaren et al., 2023). This positions EVS not only as a non-invasive biomarker of vestibular integrity but also as a promising therapeutic tool for mitigating balance impairments in aging populations (King et al., 2025).

### Long-latency/transcortical reflexes

In addition to spinal circuits, the soleus demonstrates long-latency reflexes (LLR), which occur 50–100 m after a perturbation. LLRs are slower than spinal reflexes, and involve supraspinal processing in the primary motor cortex, supplementary motor area, and cerebellum, enabling flexible, context-dependent corrections (Deligiannis et al., 2024). Unlike short-latency reflexes, which are relatively stereotyped, LLRs are highly adaptable, modulated by postural threat, surface stability, and cognitive load (Deligiannis et al., 2024). This flexibility allows the soleus activity to be adjusted in real time to changing task demands. With aging, LLRs in the soleus become delayed, smaller in amplitude, and less well modulated, potentially reflecting slowed conduction, reduced cortical excitability, and impaired sensorimotor integration (Deligiannis et al., 2024; Papegaaij et al., 2014). These deficits may limit the ability to generate rapid, context-specific corrections, contributing to instability and increased fall risk in older adults.

Collectively, these reflex pathways position the soleus as an important reflexive stabilizer, uniquely suited to maintain standing balance through high afferent coupling, tonic low-level activation, and multilevel reflex control. With aging, losses in sensitivity, excitability, and adaptability across these reflex pathways weaken the soleus’ stabilizer role. These reflex pathways provide a modifiable target for fall prevention strategies in older adults.

## Discussion

**FIGURE 1:**
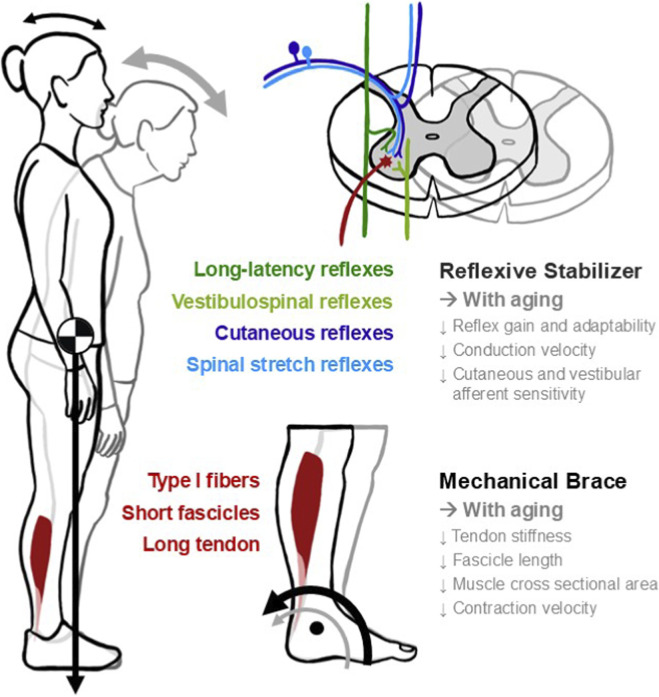
Neuromechanics of the soleus in quiet stance and aging. Schematic overview illustrating how the soleus functions as both a mechanical brace and reflexive stabilizer of standing balance. With the body’s center of mass (COM) lying anterior to the ankle, a continuous plantarflexor moment from the soleus counteracts the forward torque (black arrow: young adult; gray arrow: older adult). The soleus muscle-tendon unit, characterized by a predominance of type I fibers, short fascicles, and long compliant Achilles tendon, provide fatigue-resistant torque and buffers small perturbations. Neural control of soleus activity arises from convergent reflex pathways including spinal stretch (light blue), cutaneous (dark blue), vestibulospinal (light green), and long-latency/transcortical loops (dark green). Bulleted lists summarize age-related changes. Reflexive side: decreased reflex gain and adaptability, slower conduction, and reduced cutaneous and vestibular afferent sensitivity. Mechanical side: reduced tendon stiffness, shorter fascicle length, smaller muscle cross-sectional area, and slower contraction velocity.

In this mini-review, we advance the thesis that the soleus plays an outsized role in standing balance due to its unique mechanical and reflexive properties. These properties deteriorate with age, narrowing stability margins during quiet stance reducing the capacity to respond to perturbations (Figure 1). Framing the soleus as both a mechanical brace and a reflexive stabilizer highlights that balance control depends on the combination of rate-sensitive torque capacity, operating point of the muscle force-length curve, and task-dependent scaling of multi-sensory reflex pathways.

On the mechanical side, the soleus’ short, fatigue resistant fibers in series with the long Achilles tendon, sets an operating point that favors low-frequency torque control and energy buffering during small, continuous perturbations of quiet stance (Loram et al., 2005). The compliant Achilles tendon buffers small movements, while lengthening electromechanical delays and shifting the timing of reflex-driven corrections to later phases in sway (Cronin et al., 2013). This mechanical filtering reduces the need for strong corrective reflexes, preventing overcorrections to small perturbations. As a result, the soleus mechanically filters high-frequency noise and braces the ankle, enabling the nervous system to apply slower, adaptive corrections. Prolonged walking can induce short-term structural changes in the Achilles tendon (Cronin et al., 2009), which also may alter force transfer between muscle fascicles to the skeleton via tendons, further affecting the afferent output of muscle spindles and/or Golgi tendon organs (GTO). Age-related reductions in Achilles tendon stiffness may also alter spindle and GTO responses, elevating the risk of falling. While these passive mechanical features define the soleus as a stabilizing brace, the maintenance of postural stability also depends on active muscle control.

### Fall prevention vs. fall recovery

The soleus’ slow-twitch and tonic recruitment make it uniquely valuable for fall prevention, while the larger more mixed-fiber gastrocnemii contribute disproportionately to fall recovery (Edgerton et al., 1975; Han and Yang, 2015). More evidence in needed to directly link soleus function to fall reduction; however, fall risk is linked to poor postural control (Howcroft et al., 2017). Muscle power training, therefore, is important for maintaining reactive recovery over the lifespan, but has limited influence on quiet-stance sway (Tavakkoli et al., 2021; Pijnappels et al., 2008). The concurrent training of strength and endurance is likely the best approach to maximize fall prevention and recovery.

### Balancing tendon stiffness with compliance

Tendon stiffness dictates how muscle forces are transmitted and joint torques produced. Increased stiffness may improve RTD but reduce buffering capacity. We argue that the soleus operates in a window of optimal stiffness (Lichtwark and Wilson, 2007), compliant enough to buffer small, high frequency sways, while allowing forces to be appropriately transmitted through the muscle-fascial system. More work is needed to test interventions that increase tendon stiffness, to test the impact on stability during quiet stance and perturbations.

### Tuning reflex gain

An increase in reflex gain shifts the balance of postural control toward faster, more automatic spinal mechanisms, reducing the relative contribution of supraspinal centers (Zehr and Stein, 1999). High gain is useful for rapid stabilization, but at the cost of context-appropriate flexibility and energy efficiency (Nielsen et al., 1993; Horslen et al., 2018). Older adults often show both smaller responses and poorer modulation (Mynark and Koceja, 2002; Peters et al., 2016b; Papegaaij et al., 2014). Balance interventions should therefore target adaptive tuning of reflex gain, emphasizing training that improves context-specific modulation rather than simply increasing the reflex amplitude. Approaches such as perturbation training, dual-tasking balance training, and multi-sensory paradigms can enhance the nervous system’s ability to appropriately scale reflex responses across sensory and postural contexts (Taube et al., 2008; Thompson et al., 2009; McLaren et al., 2023). Few studies have linked individual soleus reflex excitability difference with fall risk, highlighting an important area for future research.

## Conclusion

The soleus is uniquely suited for sustaining postural stability due to its fatigue-resistant profile, large cross-sectional area, and strong reflex connectivity. Aging degrades both mechanical (tendon stiffness, RTD) and neural (reflex modulation, conduction speed) features, and exacerbates antagonist muscle fatigue, diminishing both the soleus’ stabilizing role as well as active joint stiffness through agonist-antagonist co-contraction. Although confirmatory studies linking soleus function to fall risk are still needed, preserving soleus and tibialis anterior function through targeted neuromuscular training remains a promising and underappreciated strategy for improving postural control, and potentially reducing falls in older adults, but studies confirming this hypothesis are warranted.
